# Moving Forward With Integrated Care: The Use of Realist Approaches to Understand What Works, How, for Whom and Under Which Circumstances

**DOI:** 10.3389/phrs.2022.1605082

**Published:** 2022-08-05

**Authors:** Isabelle Peytremann-Bridevaux, Maura MacPhee

**Affiliations:** ^1^ Centre for Primary Care and Public Health (Unisante), University of Lausanne, Lausanne, Switzerland; ^2^ School of Nursing, University of British Columbia-Vancouver, Vancouver, BC, Canada

**Keywords:** integrated care, complex interventions, realist methods, literature synthesis, evaluation

The purpose of our commentary is to raise the profile of alternate methodological approaches, specifically realist approaches, for advancing knowledge of and practice in healthcare innovations for diverse patient populations. We will use the example of literature reviews on integrated care for patients with chronic obstructive pulmonary disease (COPD).

Chronic diseases, such as COPD, have created significant burdens on healthcare systems. One evidence-informed approach to chronic disease management is integrated care. Implemented by healthcare systems to overcome care fragmentation, it has been shown to bring benefits to patients, at least in some healthcare dimensions.

Fifteen years ago, the first systematic reviews on integrated care for COPD patients were published [1–3]. Despite limitations and marginal differences between their results, all reviews presented evidence of decreased hospitalizations, improved quality-of-life and improved exercise capacity for COPD patients receiving integrated care. Whereas some authors advocated for higher quality trials [1], some concluded that effectiveness was difficult to ascertain with heterogeneity of patient populations, environmental factors, interventions and measures [3]; others called for research targeting a better understanding of contextual factors influencing intended outcomes [2].

End of 2021, a Cochrane review update on integrated care for COPD patients was published [4]. While doubling the number of randomized controlled studies (RCTs) meeting the eligibility criteria, this review confirmed results from earlier reviews with more data and better precision: integrated care results in decreased hospitalizations and increased quality of life and exercise capacity for COPD patients. This recent and rigorous Cochrane review [4] added limited new information, however. Acknowledging that “one size does not fit all,” authors made recommendations similar to previous ones: rigorously conducted trials and reliable measurement of outcomes are needed to minimize bias; the effects of context must be explored; pragmatic RCTs that include process and outcomes measures and qualitative assessments should be employed to better understand the findings in complex healthcare environments.

After more than a 15-year span of time, the current body of research cannot inform decisions on allocation of limited healthcare resources: what works, how, for whom and under which circumstances with respect to integrated care for COPD patients. Further research remains required to identify both key integrated care components and their optimal combinations, in order to benefit to specific patient groups.

Realist approaches, designed to answer “black box” questions, have become an increasingly utilized methodological approach for evaluating complex healthcare interventions [5]. Therefore, they can be used to understand how integrated care works, under what circumstances and for which patient populations. Indeed, realist approaches could have helped address unexpected outcomes in one US-based Veterans’ Administration multi-centric RCT where a COPD chronic management program led to more deaths in the intervention arm [6]. Realist reviews yield testable explanations of what should work for specific populations (e.g., COPD patients in integrated care), highlighting potentially effective interventions that can be further evaluated using primary data (e.g., realist evaluation approaches). They are typically based on secondary data from the peer-reviewed and grey literature, and standardized protocols are used and included documents are thoroughly vetted for relevance and rigor [7].

As an example of how realist approaches can help unpack “black box” questions, we will examine the Kastner et al. systematic review [8] conducted alongside a realist review [9] to evaluate the effectiveness of chronic disease management (CDM) tools on optimal illness management for elderly adults with multi-comorbidities. The systematic review and meta-analysis concluded that older adults with specific combinations of comorbidities (e.g., cardiovascular disease, depression, COPD) can benefit from integrated care to enhance their health status, decrease depressive symptoms and improve use of mental health services [9]. The realist review complementing the systematic review and meta-analysis findings explored contextual factors and underlying mechanisms associated with intended outcomes [10]. More specifically, this realist review uncovered how providers and patients focused on different symptoms, with different aims that compromise treatment adherence. For example, whereas patients focused on symptoms related to quality of life, providers focused on those symptoms related to morbidity and mortality. Based on both review approaches, systematic/meta-analysis and realist, Kastner et al. developed a pragmatic logic model and a testable program theory with specific explanations and recommendations for direct uptake by decision-makers, clinicians and service users [10].

Realist approaches can also examine interventions within and across systems levels. This cannot be done in RCTs since one of their shortcomings is their inability to examine complex interventions at more than one systems level, given the heterogeneity of variables and the inability to control for them. A recent example is a combined systematic review and rapid realist review of mental health interventions for individuals with chronic comorbidities during the COVID-19 pandemic [11]. Whereas documents from the systematic review were initially used to guide further searches for the realist review, the realist review yielded testable, evidence-based explanations for what works for whom under what circumstances at micro (individuals and families), meso (community, primary care providers, non-profits) and macro (government, policy-makers) levels.

Another criticism of RCTs is their strong focus on internal validity, rather than implementation, spread and sustainability. The above-mentioned realist review [8] helped identify specific integrated care approaches that optimized patient outcomes, including team-based care, disease management programs and case management models, that have supporting evidence for broader reach and sustainability. This review provided integrated care exemplars for adoption in primary healthcare settings that serve patients with chronic co-morbidities.

Finally, realist approaches can help build evaluation frameworks, such as the one of Smeets et al., who recently created a realist evaluation framework for a community-based integrated care program serving chronically ill individuals in the Netherlands [5]. First, authors created a testable, hypothetical model of the program known in realist terms as a “program theory.” Then, their review included scientific studies, policy and practice documents and expert practitioner consultations. While the ultimate goal of this work is to guide eventual country-wide spread of the program, the developed framework of testable hypotheses will guide the evaluation of a Dutch pilot and help pinpoint specific contextual factors associated with program success.

It is time for healthcare researchers studying complex healthcare interventions and programs such as integrated care, to use methodological innovations. Evaluation of complex interventions needs to go beyond efficacy and effectiveness and strive towards theory-based or system perspectives, where necessary or appropriate [12]. Systematic reviews and RCTs help us determine intervention causality. In combination with realist approaches, we can extend our understanding of why and how interventions work for specific populations, and under what circumstances.
